# Hybrid Delivery of Mindfulness Meditation and Perceived Stress in Pediatric Resident Physicians: A Randomized Clinical Trial of In-Person and Digital Mindfulness Meditation

**DOI:** 10.1007/s10880-022-09896-3

**Published:** 2022-07-01

**Authors:** Denise R. Purdie, Myke Federman, Alan Chin, Diana Winston, Brenda Bursch, Richard Olmstead, Yonca Bulut, Michael R. Irwin

**Affiliations:** 1 Department of Pediatrics, David Geffen School of Medicine at UCLA, Los Angeles, CA USA; 2 Department of Pediatrics, Irvine School of Medicine, University of California, Irvine, CA USA; 3 Department of Psychiatry & Biobehavioral Sciences, David Geffen School of Medicine at UCLA, 760 Westwood Plaza, Semel 48-241, Los Angeles, CA 90024-1759 USA

**Keywords:** Resident education, Well-being, Stress, Burnout, Mindfulness, Digital

## Abstract

Physicians are experiencing epidemic levels of work-related stress and burnout. Determine efficacy of mindfulness meditation delivered as a hybrid (in-person and digital) format to reduce perceived stress in pediatric residents. Pediatric residents (*n* = 66) were block randomized to a hybrid Mindful Awareness Practices (MAPs) intervention, comprised of one in-person 60–min session and 6-week access to a digitally delivered MAPs curriculum (*n* = 27) or wait-list control (*n* = 39). Perceived Stress Scale (PSS) was administered at baseline and post-intervention as the primary outcome measure. A priori secondary outcomes were measured using the Abbreviated Maslach Burnout Inventory-9, Beck Depression Inventory, Beck Anxiety Inventory, UCLA Loneliness Scale, and Pittsburgh Sleep Quality Index. After the first session, 58% participated at least one digital session (*M* = 2.0; *SD* = 1.3). MAPs participants showed significant decrease in PSS compared to controls, with between-group mean difference of 2.20 (95% CI 0.47–3.93) at post-intervention (effect size 0.91; 0.19–1.62). No secondary outcome group differences were detected. Exposure to a hybrid mindfulness intervention was associated with improvement in perceived stress among pediatric residents.

*Trial Registration*: NCT03613441.

## Introduction

The silent suffering of physicians and other caregivers due to stress, burnout and depression is rising to epidemic proportions in many specialties. (Nasca, 2016).

The World Health Organization defines burnout (characterized by “feelings of energy depletion or exhaustion; increased mental distance from one’s job, or feelings of negativism or cynicism related to one’s job; and reduced professional efficacy”) as a failure of stress management in the workplace (2019). The Accreditation Council for Graduate Medical Education (ACGME) has focused its recent attention on physician wellness as it is increasingly clear clinician burnout needs to be tackled early in professional development. By the end of medical school, nearly half of medical school graduates endorse symptoms of stress and burnout and over a third endorse symptoms of depression (Brazeau et al., 2014; Dyrbye & Shanafelt, 2016; Dyrbye et al., 2006). This finding is striking because medical students begin their training with similar or better mental health than age-matched peers (Chaukos et al., 2017). Research focused on pediatric residents similarly reveals that they frequently experience burnout and mental health symptoms (Baer et al., 2017; Cellini et al., 2017; Kemper et al., 2020; McKinley et al., 2017; Olsen et al., 2015; Pantaleoni et al., 2014). Stress has been found to be a potent risk factor for psychological symptoms (i.e., depression, anxiety) and emotional exhaustion in more than 50% of resident physicians across all specialties (Dyrbye & Shanafelt, 2016; Dyrbye et al., 2011, 2014; Slavich & Irwin, 2014).

In addition to depression and burnout, stress in resident physicians is also associated with substance dependence and suicide (Dyrbye et al., 2011; Oreskovich et al., 2015; van der Heijden et al., 2008; Wallace et al., 2009), suboptimal patient care (i.e., treatment and medication errors, failure to adhere to best practices, reduced attentiveness to patients, and failure to fully discuss treatment options or answer patient questions) (Dyrbye & Shanafelt, 2016; Fahrenkopf et al., 2008; Matsuo et al., 2021; Shanafelt et al., 2002; West et al., 2009), an erosion of professionalism (Dyrbye et al., 2014), and career dissatisfaction leading to increased likelihood of abandoning medicine (Becker et al., 2006). Although less well studied among physicians, loneliness has also been linked to burnout among internal medicine residents (Shapiro et al., 2015). Increased social support has been shown to reduce loneliness and subsequently reduce burnout among trainees (Rogers et al., 2016). Finally, high levels of perceived stress are associated with poor sleep and fatigue (Arafat & Kabir, 2017; Levey, 2001). Residents suffering from sleep deprivation have an increased risk of medical errors, injuries, alcohol and drug use, and conflict with other health professionals (Baldwin & Daugherty, 2004).

With growing recognition that burnout is a complex construct, there is an emerging understanding that both individual-focused as well as structural or organizational strategies can result in reductions in symptoms of burnout among healthcare professional as reported in recent meta-analyses (Spinelli et al., 2019; West et al., 2016). The most commonly studied individual-focused interventions have involved mindfulness or stress management approaches. Mindfulness interventions train one in the systematic practice of attending to moment-by-moment experiences, thoughts, and emotions from a nonjudgmental perspective (Black et al., 2015; Lamothe et al., 2018). Mindfulness interventions have been demonstrated to have moderate effects on perceived stress (the degree to which life situations are appraised as stressful) and to reduce symptoms of insomnia, loneliness, depression, and anxiety (Chen et al., 2020; Goyal et al., 2014; Oman et al., 2006; Romcevich et al., 2018; Spinelli et al., 2019; Thimmapuram et al., 2021). Among pediatric resident physicians, mindfulness has been associated with lower stress, decreased burnout, and greater confidence in providing compassionate care (Kemper et al., 2019; Reed et al., 2018). However, the few existing randomized controlled trials have demonstrated mixed support for utility of mindfulness skills in reducing burnout among resident physicians (Fraiman et al., 2022; Ireland et al., 2017; Lebares et al., 2018; Verweij et al., 2018). Additionally, only one small pilot study, to our knowledge, has evaluated whether or not hybrid (in-person and digital) delivery of mindfulness reduces perceived stress in resident physicians (Romcevich et al., 2018). They detected a significant decrease in perceived stress as well as improved resilience and decreased levels of burnout.

We conducted a randomized controlled clinical trial to examine the effect of a low-cost, community-accessible mindfulness-based intervention, known as Mindful Awareness Practices (MAPs) (Black et al., 2015) on perceived stress in pediatric resident physicians. In contrast to prior studies targeting physicians in training which have been delivered in person (Ireland et al., 2017; Lebares et al., 2018; Verweij et al., 2018), we offered mindfulness training in a hybrid format, with the initial session in person followed by a digital format. As compared to a wait-list control, the research-based curriculum, MAPs, was hypothesized to confer improvements on the primary outcome, perceived stress. Perceived stress was chosen as the primary outcome because, based on the published research to date, it appears to be an important precursor to a variety of negative outcomes and potentially more sensitive to mindfulness techniques than burnout, which was also measured. Additionally, it was hypothesized that hybrid MAPs would lead to improvements in depression, anxiety, loneliness, and sleep quality based on prior evidence (Black et al., 2015; Bower et al., 2015; Creswell et al., 2012; Lopez-Maya et al., 2019).

## Methods

### Trial Design and Randomization

This was a single-site, parallel-group, randomized control trial. Prior to the start of the trial, residents were assigned to various geographic training sites within the UCLA pediatric residency training program without interaction with study investigators. Based on these geographic assignments, two blocks were constructed and used as the unit of randomization. One block was allocated 1:1 to MAPs and the other block was allocated to wait-list control using computerized random number generation before the start of the trial. The University of California, Los Angeles institutional review board approved all procedures. Participants provided written informed consent.

### Setting and Participants

The study was conducted at the University of California, Los Angeles from September 2017 to March 2018. Inclusion criteria were status as resident physician in the pediatric and medicine-pediatric program at the UCLA Mattel Children’s Hospital. All participants spoke English and had not received prior training in MAPs.

### Procedures

Participants who consented to study procedures completed assessments at baseline prior to the intervention and immediately post-intervention. Participants were blinded to the randomization until the start of the intervention. Data collectors were unaware of group assignment and were instructed to treat all participants in the same manner. Additionally, self-report questionnaires were anonymous, matched via a participant-derived identifier number, and completed privately by participants. After study completion, the MAPs intervention was opened to the control condition, according to the wait-list design.

### Interventions

Mindful Awareness Practices (MAPs) is a mindfulness-based intervention developed by Diana Winston at UCLA’s Mindful Awareness Research Center (MARC). MAPs is a weekly 2-h, 6-session group-based curriculum in mindfulness meditation that is widely available in-person and online (http://marc.ucla.edu). Mindfulness exercises include mindful breathing, mindful sitting, mindful eating, mindful listening, appreciation meditation, friendly or loving-kindness meditation, mindful walking, and mindful movement as previously described (Black et al., 2015). MAPs trains one in the systematic practice of attending to moment-by-moment experiences, thoughts, and emotions from a nonjudgmental perspective (Brown & Ryan, 2003) is similar to Mindfulness-Based Stress Reduction (MBSR), yet is more accessible by not requiring a day-long retreat or Hatha yoga.

Given the time constraints of resident physicians, the MAPs course was modified with the first session being teacher-delivered, and the five remaining sessions being delivered in a digital format, as self-study sessions via a secure mobile app. The in-person session was thought to be important to give the participants a guided, teacher taught experience of mindfulness meditation. Prior studies using a digital-only format of mindfulness have found benefit for depressive, anxiety, and insomnia symptoms, although adherence rates are low (Huberty, Green, et al., 2021; Huberty, Green, et al., 2021; Huberty, Puzia, et al., 2021; Huberty, Puzia, et al., 2021). The app was developed as a participatory mobile health framework using a web application platform called UCLA CHORUS (Arevian, Bell, et al., 2018; Arevian, O’Hora, et al., 2018) which is identical to the content of online sessions available through UCLA’s MARC and the app “UCLA Mindful”.

Diana Winston, who originated the MAPs curriculum and has more than 20 years of teaching experience in mindfulness, delivered the in-person session and oversaw fidelity of the mobile app relative to the previously developed online MAPs course available through MARC (Bower et al., 2015; Lopez-Maya et al., 2019). Each session introduced the participants to another aspect of mindfulness meditation as per the MAPs course. The participants were then asked to complete 5–20 min daily of mindfulness practice using the guided meditations available through the app (with a weekly increase in practice by 5 min). A trained mindfulness educator was also available for questions via an anonymous discussion board during the intervention period.

The wait-list control was informed of the MAPs intervention at the time of consent and were eligible to enroll in the MAPs intervention in the format described above following study completion.

### Outcomes and Assessments

Self-report questionnaires were administered before and after the intervention with all post-intervention assessments completed within 2 weeks after the intervention (week 8).

The primary outcome was perceived stress, which was measured using the Perceived Stress Scale (PSS). This is a widely validated 14-item self-report questionnaire and measures the degree to which situations in one’s life are appraised as stressful (Cohen et al, 1983). Participants are asked to indicate how often they felt or thought a certain way on a 5-point Likert scale (for example, “In the last month, how often have your felt that you were unable to control the important things in your life?”. Scores may range from 0 to 40 with higher scores indicating higher levels of perceived stress. Because the PSS is not a diagnostic instrument with an established clinical threshold, minimal meaningful change was defined using relative change in the PSS (i.e., percentage change from baseline) and the optimal threshold of 28%, a cut-point validated for work-related stress by the external anchor of Patient’s Global Impression of Change (Eskildsen et al., 2015). PSS was specified as the primary outcome in the protocol to the institutional review board prior to enrollment of participants.

Secondary outcomes, which were also planned and a priori, were those thought to be related to the perceived stress of resident physicians including the reflected the causes and consequences of psychological distress suffered by physicians and included multiple well-validated measures: Abbreviated Maslach Burnout Inventory-9 (MBI) (Maslach et al., 1996), Beck Depression Inventory (BDI) (Beck et al., 1996), Beck Anxiety Inventory (BAI) (Beck & Steer, 1990), UCLA Loneliness Scale (Russell et al., 1980), and Pittsburgh Sleep Quality Index (PSQI) (Buysse et al., 1989; Cole et al., 2006).

Adherence to digital MAPs and mindfulness practice was evaluated at post-intervention, using previously reported methods (Black et al., 2015; Bower et al., 2015; Lopez-Maya et al., 2019), by self-report of number of completed sessions, number of days with at least 5 min of mindfulness practice, and number of minutes of practice per day in the last week, which was summed to create total number of practice minutes per week.

Demographic information was obtained at baseline including age, sex, ethnicity, marital status, and residency training year.

### Sample Size

A power analysis was conducted in G × Power (http://www.gpower.hhu.de/en.html). Based on previous meta-analytic trials and mean treatment effect (*d* = .4, a medium effect size), an estimated final total sample size of 52 participants provided statistical power of 80% (a = .05) to detect a between-group effect for psychological stress, i.e., primary outcome perceived stress (Black et al., 2015). An attrition rate of 10% was anticipated, making our target enrollment 29 participants per group.

### Statistical Analysis

Between-group change in the mean perceived stress at post-intervention was the primary outcome in the intent-to-treat (ITT) population (i.e., participants randomized and allocated to the intervention with attendance at first in-person session). Analyses were performed in SAS software, version 9.4 (SAS Inc., Cary, NC, USA). Between-group contrasts in outcomes across the intervention period were tested using generalized linear mixed modeling (MIXED command with full information maximum likelihood estimation to allow for analysis of participants with missing data) with pairwise comparisons, adjusted baseline levels of the outcome. Estimated mean differences and effect sizes (Cohen *d* with Hedges bias correction for small sample size) with their 95% confidence intervals are provided. Exploratory analyses were also conducted to explore whether change in the primary outcome, PSS, achieved a threshold for minimally clinically important change and whether this change differed between MAPs vs control. The proportion of participants who achieved this relative change in the PSS in the MAPs vs control was tested by Likelihood ratio test.

## Results

### Participant Flow and Characteristics

**Fig. 1:**
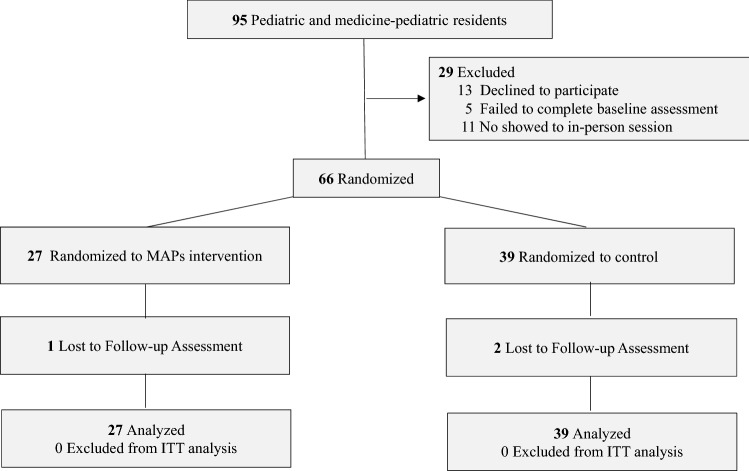
Consolidated Standards of Reporting Trials (CONSORT) flow diagram of single-site, parallel-group randomized clinical trial of MAPs compared with wait-list control for perceived stress in pediatric resident physicians. *ITT* intent-to-treat, *MAPs* Mindful Awareness Practices, *control* wait-list control. Reasons for declining to participate were time limitations

Participant flow through enrollment, randomization, follow-up, and analysis phases of the trial is shown in Fig. 1. Eligible participants who agreed to participate and completed the baseline assessment were randomized by block; 27 were randomized and allocated to the hybrid MAPs, and 39 were assigned to wait-list controls. Among participants allocated to MAPs, all attended the first in-person session. However, only 58% of those allocated to MAPs participated in any of the digital sessions, with an overall completion of 2.0 digital sessions (SD, 1.3) in the total group. The mean number of days of self-directed mindfulness practice per week was 2.2 days (SD, 1.9) and mean number of minutes per day was 7.0 min (SD, 12.8). Minutes of self-directed mindfulness practice per week ranged from 0 to 240 min, with 53% (*n* = 14) practicing at least 10 min per week.

Table 1 lists summary descriptive statistics for the study groups at baseline. None of the baseline variables differed across groups. The baseline raw mean scores of self-reported stress (MAPs: 26.4 [SD, 5.2] vs control: 25.8 [SD, 6.2] showed no significant differences between study groups. Of note, these means reflect moderate levels of perceived stress in this residency population. Table 2 displays intent-to-treat model-derived estimates for primary and secondary outcome measures, revealing moderately elevated levels of emotional exhaustion and frequent feelings of loneliness, low-range severity of depression and anxiety symptoms, and sleep quality below the threshold of sleep impairment (PSQI < 5).

**Table 1:** Baseline demographic characteristics by intervention group

Variable	MAPs (*n* = 27)	Wait-list control (*n* = 39)	*p* value
*Demographic characteristics*			
Age, 25–34 y	26 (96%)	37 (100%)	.43
Female sex, No. (%)	24 (89%)	28 (74%)	.21
Married, No. (%)	22 (81%)	23 (61%)	.11
Race/ethnicity, No. (%)^a^			.24
Asian/Pacific Islander	13 (48%)	13 (34%)	
Hispanic/Latino	0	1 (3%)	
White	13 (48%)	17 (45%)	
Other	1 (4%)	7 (18%)	
Residency training year, No. (%)^b^			.11
PGY-1	13 (48%)	10 (27%)	
PGY-2	7 (26%)	8 (27%)	
PGY-3/4	7 (26%)	19 (51%)	

^a^One participant declined to answer race/ethnicity question

^b^Two participants declined to answer residency training year question

**Table 2:** Intent-to-treat model estimates for primary and secondary outcome measures

	Estimated mean (SD)					
	MAPs (*n* = 27)		Wait-list control (*n* = 39)		Value (95% CI)	
Outcome, scale range	Baseline	Post-intervention	Baseline	Post-intervention	Difference in post-intervention values	Effect size^c^
*Primary outcome*						
Perceived stress scale, 0–70^a^	25.9 (7.3)	22.4 (7.4)^d^	25.8 (6.0)	24.1 (6.1)	2.20 (0.47–3.93)	0.91 (0.19–1.62)
*Secondary outcomes*						
Maslach burnout inventory, 0–18						
Emotional exhaustion^a^	10.7 (4.8)	9.7 (4.9)	10.6 (4.0)	10.2 (4.1)	0.48 (− 0.66–1.62)	0.30 (− 0.41–1.01)
Depersonalization^a^	6.0 (4.8)	5.6 (4.7)	5.9 (4.0)	6.0 (4.1)	0.37 (− 0.77–1.51)	0.23 (− 0.48–0.94)
Personal accomplishment^b^	13.8 (3.9)	14.1 (3.9)	13.9 (3.1)	13.9 (3.3)	− 0.25 (− 1.17–0.67)	− 0.20 (− 0.92–0.52)
Beck depression inventory, 0–63^a^	6.8 (6.0)	7.2 (6.2)	6.8 (5.0)	6.4 (5.2)	− 0.80 (− 2.23–0.63)	− 0.40 (− 1.10–0.31)
Beck anxiety inventory, 0–63^a^	6.8 (7.4)	4.2 (7.5)^d^	6.1 (6.1)	4.3 (6.5)^d^	0.08 (− 1.70–1.86)	0.03 (− 0.69–0.75)
UCLA loneliness scale, 20–80^a^	37.6 (10.3)	37.2 (10.5)	37.4 (8.5)	37.9 (8.8)	0.65 (− 1.78–3.09)	0.19 (− 0.52–0.90)
Pittsburgh Sleep Quality Index, 0–21^a^	5.0 (3.2)	4.9 (3.2)	5.2 (2.7)	4.7 (2.8)	− 0.14 (− 0.90–0.63)	− 0.13 (− 0.84–0.59)

^a^Lower scores indicate improvement

^b^Higher scores indicated improvement

^c^Bias-corrected Hedges g covarying for baseline values

^d^Significant change from baseline to post-intervention at *p* < 0.05

### Primary Outcome

**Fig. 2:**
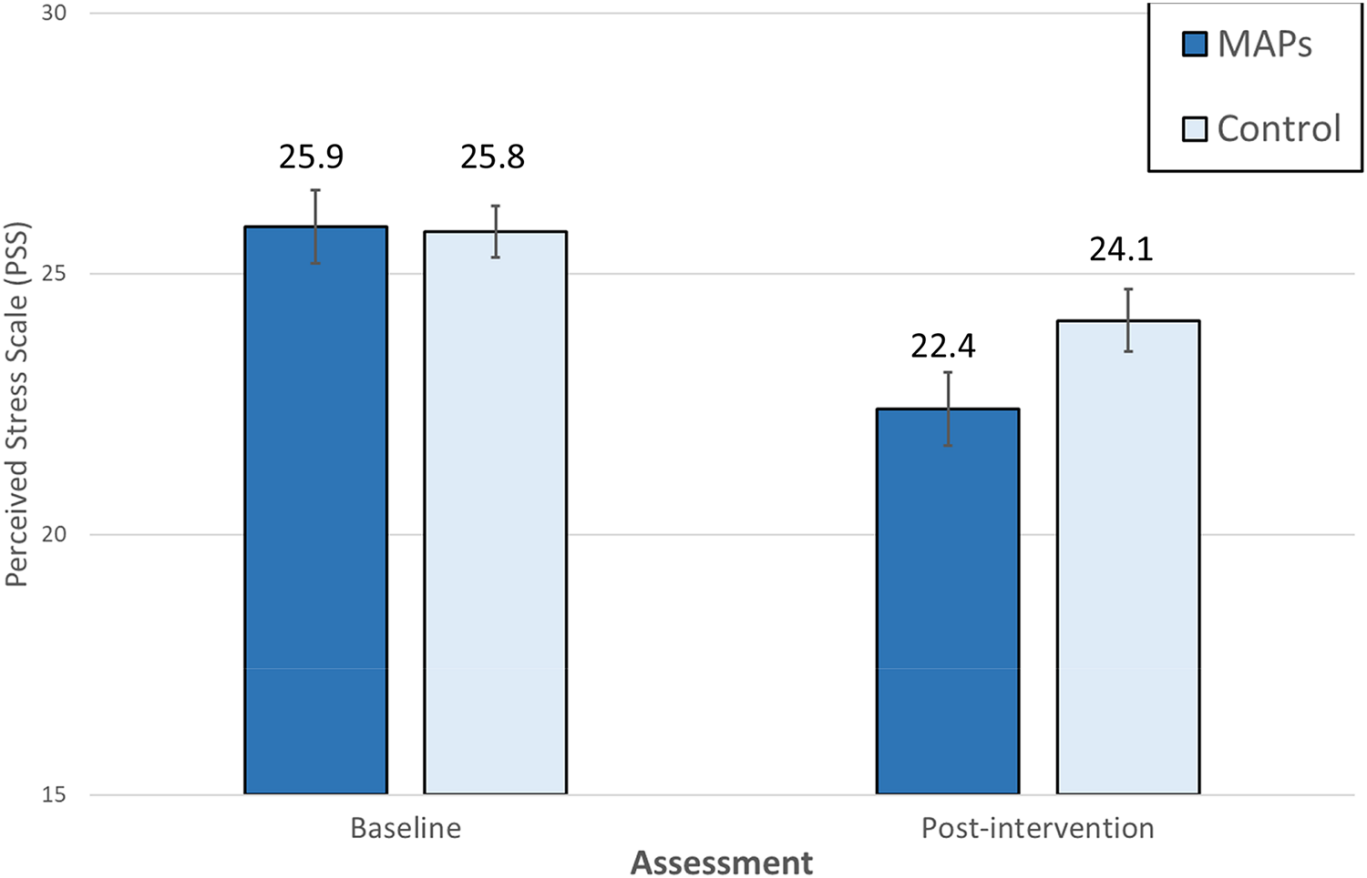
Estimated Perceive Stress Scale score at baseline and post-intervention. Data are given as means (SEs). *MAPs* Mindful Awareness Practices, *control* wait-list control. *p* = .002 for difference between groups, covarying for baseline Perceived Stress Scale score

Primary and secondary outcome ITT analyses included participants randomized and allocated to group assignment regardless of measured program adherence or missing data (*n* = 66). The Perceived Stress Scale (PSS) score improved by a mean of 3.5 in the MAPs group and by a mean of 1.7 in the control group, indicating greater improvement in the MAPs group (between-group mean difference, 2.20; 95% CI 0.47–3.93) with a large effect size 0.91 (0.19–1.62) (Table 2 and Fig. 2). At post-intervention, number of minutes of mindfulness practice negatively correlated with scores on the PSS (*r* = − 0.39, *p* = 0.05), suggesting that amount of daily mindfulness practice was associated with lower levels of perceived stress.

Exploratory analyses tested whether relative change in the primary outcome, PSS, achieved a threshold for minimally clinically important change in MAPs versus control groups. Using the optimal threshold for detection of minimal meaningful change for work-related stress (Eskildsen et al., 2015), the proportion who reported they achieved a meaningful change was 32% in MAPs vs 13% in control, but did not reach statistical significance (χ^2^ = 3.1, *p* = 0.08).

### Secondary Outcomes

Changes over time were statistically equivalent across treatment groups for the secondary outcomes including levels of burnout or symptoms of depression, anxiety, loneliness, and sleep disturbance.

Inclusion of covariates such as residency year, marital status, and ethnicity did not significantly alter the results.

## Discussion

This randomized controlled trial examined the effect of a hybrid, in-person and digital, mindfulness meditation program on perceived stress in resident physicians undergoing training in pediatrics and medicine-pediatrics. To our knowledge, this study is the first to demonstrate the efficacy of a hybrid mindfulness curriculum, delivered an in-person and digital format, to improve perceived stress relative to a wait-list control group in physicians in training. The effect size of 0.9 for improvement in perceived stress was large, which is notable given meta-analytic findings comparing various meditation treatment modalities with mean effect sizes for psychological distress of small to medium magnitude. Moreover, the effect of hybrid MAPs to improve perceived stress is especially compelling given the rather modest rates of adherence to the digital format (see limitation section below). Finally, exploratory findings suggest that a meaningful change in the PSS occurred at an over two-fold greater rate in MAPs as compared to control. While further research is needed to determine if this meaningful change construct is a valuable way to examine outcomes, these results have important preliminary implications for the value of mindfulness interventions to mitigate work-related stress of physicians in training.

The large effect of hybrid MAPs to reduce perceived stress may be due to the efficacy of this specific mindfulness curriculum, sample characteristics, demand characteristics, and/or expectancy effects. The in-person MAPs curriculum has been previously reported to be effective in improving sleep disturbance (Black et al., 2015) as well as reducing depressive symptoms (Bower et al., 2015; Lopez-Maya et al., 2019) despite limited effects of other meditation approaches on these outcomes (Goyal et al., 2014). Thus, the MAPs approach might be uniquely impactful. Because all participants had at least moderate levels of stress at baseline, they may also have been primed to benefit from the MAPs intervention. Since participants could not be blinded to the fact that they were receiving the intervention, some may have changed their actions and behaviors to become less stressed unrelated to MAPs.

The present study did not demonstrate effects on secondary outcomes of depression, anxiety, burnout, loneliness, or sleep disturbance. Thus, there are now two randomized clinical trials of mindfulness meditation with pediatric resident physicians that have failed to demonstrate an impact on burnout (Fraiman et al., 2022). This likely reflects the multiple workplace- and nonworkplace-related factors associated with burnout and the need for not only individual-based but systems-wide interventions to reduce stress. Mindfulness meditation might need to be combined with other individual-based interventions that improve motivation, communication skills, teamwork, and engagement in participatory and self-care programs; or structural changes that modify work demands, schedule, and/or shift duration (Aryankhesal et al., 2019; West et al., 2016). Further, interventions may need to be tailored to the specific environmental stressors.

Our study has some important limitations to consider. Despite delivery of mindfulness training via a convenient mobile app, measured adherence to the intervention was low. While even intermittent adherence to mobile-based apps for mindfulness is reported to improve reports of well-being (Clarke & Draper, 2019), our findings are more robust than one might expect. Although many residents did not subsequently access the digital format, it could be that the initial session was sufficient to teach the core skills to residents or served as a reminder to them to use previously learned skills. Additionally, the measured level of practice in this study was “formal practice,” however, we anecdotally discovered that participants informally practiced by using mindful awareness to be present in the moment in daily activities. This suggests that teaching residents to integrate these skills within their workflow might be more feasible than formal practice and that measurement of workflow practice (as opposed to formal practice) is needed to better describe adherence. As discussed above, expectancy effects may have also played a role in explaining our large effect size. The study was also conducted at a single institution, with participants limited to residents in Pediatrics, and with a high proportion of Asian ethnicity, which potentially limits the generalizability of the results. Resident physicians were asked to complete the mindfulness-based intervention outside of work hours, which adds to their stressful schedule and likely contributed to low adherence. Engagement of the residency training program in delivering mindfulness training or other tools to reduce psychological stress during formal work hours would likely optimize adherence and better promote resilience.

## Conclusions

Exposure to training in MAPs mindfulness meditation, delivered in person and digitally, was associated with reduced levels of perceived stress in resident physicians. Targeting work-related stress with MAPs has the potential to mitigate the development of clinician burnout and to promote physician well-being.

## Data Availability

Available.

## Abbreviations

PSS: Perceived stress scale
MBI: Abbreviated Maslach Burnout Inventory-9
BDI: Beck depression inventory
BAI: Beck anxiety inventory
PSQI: Pittsburgh Sleep Quality Index
MAPs: Mindful Awareness Practices (MAPs)

## References

[CR1] Arafat SY, Kabir R (2017). Burnout in physicians: Global and Bangladesh perspective. EC Psychol Psychiatry.

[CR2] Arevian AC, Bell D, Kretzman M, Kasari C, Narayanan S, Kesselman C, Wu S, Di Capua P, Hsu W, Keener M, Pevnick J, Wells KB, Chung B (2018). Participatory methods to support team science development for predictive analytics in health. Journal of Clinical and Translational Science.

[CR4] Arevian AC, O’Hora J, Jones F, Mango J, Jones L, Williams PG, Booker-Vaughns J, Jones A, Pulido E, Banner-Jackson D, Wells KB (2018). Participatory technology development to enhance community resilience. Ethnicity & Disease.

[CR5] Aryankhesal A, Mohammadibakhsh R, Hamidi Y, Alidoost S, Behzadifar M, Sohrabi R, Farhadi Z (2019). Interventions on reducing burnout in physicians and nurses: A systematic review. Medical Journal of the Islamic Republic of Iran.

[CR6] Baer TE, Feraco AM, Tuysuzoglu Sagalowsky S, Williams D, Litman HJ, Vinci RJ (2017). Pediatric resident burnout and attitudes toward patients. Pediatrics.

[CR7] Baldwin DC, Daugherty SR (2004). Sleep deprivation and fatigue in residency training: Results of a national survey of first-and second-year residents. Sleep.

[CR8] Beck AT, Steer RA (1990). Manual for the beck anxiety inventory.

[CR9] Beck AT, Steer RA, Brown GK (1996). Manual for the beck depression inventory-II.

[CR10] Becker JL, Milad MP, Klock SC (2006). Burnout, depression, and career satisfaction: Cross-sectional study of obstetrics and gynecology residents. American Journal of Obstetrics and Gynecology.

[CR11] Black DS, O'Reilly GA, Olmstead R, Breen EC, Irwin MR (2015). Mindfulness meditation and improvement in sleep quality and daytime impairment among older adults with sleep disturbances: A randomized clinical trial. JAMA Internal Medicine.

[CR12] Bower JE, Crosswell AD, Stanton AL, Crespi CM, Winston D, Arevalo J, Ma J, Cole SW, Ganz PA (2015). Mindfulness meditation for younger breast cancer survivors: A randomized controlled trial. Cancer.

[CR13] Brazeau CM, Shanafelt T, Durning SJ, Massie FS, Eacker A, Moutier C, Satele DV, Sloan JA, Dyrbye LN (2014). Distress among matriculating medical students relative to the general population. Academic Medicine: Journal of the Association of American Medical Colleges.

[CR14] Brown KW, Ryan RM (2003). The benefits of being present: Mindfulness and its role in psychological well-being. Journal of Personality and Social Psychology.

[CR15] Buysse DJ, Reynolds CF, Monk TH, Berman SR, Kupfer DJ (1989). The Pittsburgh Sleep Quality Index: A new instrument for psychiatric practice and research. Psychiatry Research.

[CR16] Cellini MM, Serwint JR, Chaudron LH, Baldwin CD, Blumkin AK, Szilagyi PG (2017). Availability of emotional support and mental health care for pediatric residents. Academic Pediatrics.

[CR17] Chaukos D, Chad-Friedman E, Mehta DH, Byerly L, Celik A, McCoy TH, Denninger JW (2017). Risk and resilience factors associated with resident Burnout. Academic Psychiatry: THe Journal of the American Association of Directors of Psychiatric Residency Training and the Association for Academic Psychiatry.

[CR18] Chen TL, Chang SC, Hsieh HF, Huang CY, Chuang JH, Wang HH (2020). Effects of mindfulness-based stress reduction on sleep quality and mental health for insomnia patients: A meta-analysis. Journal of Psychosomatic Research.

[CR19] Clarke J, Draper S (2019). Intermittent mindfulness practice can be beneficial, and daily practice can be harmful. An in depth, mixed methods study of the “Calm” app’s (mostly positive) effects. Internet Interventions.

[CR20] Cohen S, Kamarck T, Mermelstein R (1983). A global measure of perceived stress. Journal of Health and Social Behavior.

[CR21] Cole JC, Motivala SJ, Buysse DJ, Oxman MN, Levin MJ, Irwin MR (2006). Validation of a 3-factor scoring model for the Pittsburgh sleep quality index in older adults. Sleep.

[CR22] Creswell JD, Irwin MR, Burklund LJ, Lieberman MD, Arevalo JM, Ma J, Breen EC, Cole SW (2012). Mindfulness-Based Stress Reduction training reduces loneliness and pro-inflammatory gene expression in older adults: A small randomized controlled trial. Brain, Behavior, and Immunity.

[CR23] Dyrbye LN, Moutier C, Durning SJ, Massie FS, Power DV, Eacker A, Harper W, Thomas MR, Satele D, Sloan JA, Shanafelt TD (2011). The problems program directors inherit: Medical student distress at the time of graduation. Medical Teacher.

[CR24] Dyrbye L, Shanafelt T (2016). A narrative review on burnout experienced by medical students and residents. Medical Education.

[CR25] Dyrbye LN, Thomas MR, Shanafelt TD (2006). Systematic review of depression, anxiety, and other indicators of psychological distress among U.S. and Canadian medical students. Academic Medicine: Journal of the Association of American Medical Colleges.

[CR26] Dyrbye LN, West CP, Satele D, Boone S, Tan L, Sloan J, Shanafelt TD (2014). Burnout among U.S. medical students, residents, and early career physicians relative to the general U.S. population. Academic Medicine: Journal of the Association of American Medical Colleges.

[CR27] Eskildsen A, Dalgaard VL, Nielsen KJ, Andersen JH, Zachariae R, Olsen LR, Jørgensen A, Christiansen DH (2015). Cross-cultural adaptation and validation of the Danish consensus version of the 10-item Perceived Stress Scale. Scandinavian Journal of Work, Environment & Health.

[CR28] Fahrenkopf AM, Sectish TC, Barger LK, Sharek PJ, Lewin D, Chiang VW, Edwards S, Wiedermann BL, Landrigan CP (2008). Rates of medication errors among depressed and burnt out residents: Prospective cohort study. BMJ (Clinical Research Ed.).

[CR29] Fraiman YS, Cheston CC, Cabral HJ, Allen C, Asnes AG, Barrett JT, Batra M, Bernstein W, Bleeker T, Dietz PM, Lewis J, Li ST, Ma TM, Mahan JD, Michelson CD, Poynter SE, Vining MA, Watson K, Sox CM (2022). Effect of a novel mindfulness curriculum on burnout during pediatric internship: A cluster randomized clinical trial. JAMA Pediatrics.

[CR31] Goyal M, Singh S, Sibinga EM, Gould NF, Rowland-Seymour A, Sharma R, Berger Z, Sleicher D, Maron DD, Shihab HM, Ranasinghe PD, Linn S, Saha S, Bass EB, Haythornthwaite JA (2014). Meditation programs for psychological stress and well-being: A systematic review and meta-analysis. JAMA Internal Medicine.

[CR32] Huberty JL, Green J, Puzia ME, Larkey L, Laird B, Vranceanu AM, Vlisides-Henry R, Irwin MR (2021). Testing a mindfulness meditation mobile app for the treatment of sleep-related symptoms in adults with sleep disturbance: A randomized controlled trial. PLoS ONE.

[CR33] Huberty J, Puzia ME, Green J, Vlisides-Henry RD, Larkey L, Irwin MR, Vranceanu AM (2021). A mindfulness meditation mobile app improves depression and anxiety in adults with sleep disturbance: Analysis from a randomized controlled trial. General Hospital Psychiatry.

[CR34] Ireland MJ, Clough B, Gill K, Langan F, O'Connor A, Spencer L (2017). A randomized controlled trial of mindfulness to reduce stress and burnout among intern medical practitioners. Medical Teacher.

[CR35] Kemper KJ, McClafferty H, Wilson PM, Serwint JR, Batra M, Mahan JD, Schubert CJ, Staples BB, Schwartz A, Pediatric Resident Burnout-Resilience Study Consortium (2019). Do mindfulness and self-compassion predict burnout in pediatric residents?. Academic Medicine: Journal of the Association of American Medical Colleges.

[CR36] Kemper KJ, Schwartz A, Wilson PM, Mahan JD, Schubert CJ, Staples BB, McClafferty H, Serwint JR, Batra M, Pediatric Resident Burnout-Resilience Study Consortium (2020). Burnout in pediatric residents: Three years of national survey data. Pediatrics.

[CR37] Lamothe M, McDuff P, Pastore YD, Duval M, Sultan S (2018). Developing professional caregivers’ empathy and emotional competencies through mindfulness-based stress reduction (MBSR): Results of two proof-of-concept studies. British Medical Journal Open.

[CR38] Lebares CC, Hershberger AO, Guvva EV, Desai A, Mitchell J, Shen W, Reilly LM, Delucchi KL, O'Sullivan PS, Ascher NL, Harris HW (2018). Feasibility of formal mindfulness-based stress-resilience training among surgery interns: A randomized clinical trial. JAMA Surgery.

[CR39] Levey RE (2001). Sources of stress for residents and recommendations for programs to assist them. Academic Medicine.

[CR40] Lopez-Maya E, Olmstead R, Irwin MR (2019). Mindfulness meditation and improvement in depressive symptoms among Spanish- and English speaking adults: A randomized, controlled, comparative efficacy trial. PLoS ONE.

[CR41] Maslach, C., Jackson, S. E., & Leiter, M. P. (1996). Maslach burnout inventory manual. Third edition. Mountain View, California. Scarecrow Education

[CR42] Matsuo T, Takahashi O, Kitaoka K, Arioka H, Kobayashi D (2021). Resident burnout and work environment. Internal Medicine.

[CR43] McKinley TF, Boland KA, Mahan JD (2017). Burnout and interventions in pediatric residency: A literature review. Burnout Research.

[CR45] Nasca TJ (2016). Introduction to the CLER national report of findings 2016. Journal of Graduate Medical Education.

[CR46] Olson K, Kemper KJ, Mahan JD (2015). What factors promote resilience and protect against burnout in first-year pediatric and medicine-pediatric residents?. Journal of Evidence-Based Complementary & Alternative Medicine.

[CR47] Oman D, Hedberg J, Thoresen CE (2006). Passage meditation reduces perceived stress in health professionals: A randomized, controlled trial. Journal of Consulting and Clinical Psychology.

[CR48] Oreskovich MR, Shanafelt T, Dyrbye LN, Tan L, Sotile W, Satele D, West CP, Sloan J, Boone S (2015). The prevalence of substance use disorders in American physicians. The American Journal on Addictions.

[CR49] Pantaleoni JL, Augustine EM, Sourkes BM, Bachrach LK (2014). Burnout in pediatric residents over a 2-year period: A longitudinal study. Academic Pediatrics.

[CR50] Reed S, Kemper KJ, Schwartz A, Batra M, Staples BB, Serwint JR, McClafferty H, Schubert CJ, Wilson PM, Rakowsky A, Chase M, Mahan JD (2018). Variability of burnout and stress measures in pediatric residents: An exploratory single-center study from the pediatric resident burnout-resilience study consortium. Journal of Evidence-Based Integrative Medicine.

[CR51] Rogers E, Polonijo AN, Carpiano RM (2016). Getting by with a little help from friends and colleagues: Testing how residents’ social support networks affect loneliness and burnout. Canadian Family Physician.

[CR52] Romcevich LE, Reed S, Flowers SR, Kemper KJ, Mahan JD (2018). Mind-body skills training for resident wellness: A pilot study of a brief mindfulness intervention. Journal of Medical Education and Curricular Development.

[CR54] Russell D, Peplau LA, Cutrona CE (1980). The revised UCLA Loneliness Scale: Concurrent and discriminant validity evidence. Journal of Personality and Social Psychology.

[CR55] Shanafelt TD, Bradley KA, Wipf JE, Back AL (2002). Burnout and self-reported patient care in an internal medicine residency program. Annals of Internal Medicine.

[CR56] Shapiro J, Zhang B, Warm EJ (2015). Residency as a social network: Burnout, loneliness, and social network centrality. Journal of Graduate Medical Education.

[CR57] Slavich GM, Irwin MR (2014). From stress to inflammation and major depressive disorder: A social signal transduction theory of depression. Psychological Bulletin.

[CR58] Spinelli C, Wisener M, Khoury B (2019). Mindfulness training for healthcare professionals and trainees: A meta-analysis of randomized controlled trials. Journal of Psychosomatic Research.

[CR59] Thimmapuram J, Pargament R, Bell T, Schurk H, Madhusudhan DK (2021). Heartfulness meditation improves loneliness and sleep in physicians and advance practice providers during COVID-19 pandemic. Hospital Practice.

[CR60] van der Heijden F, Dillingh G, Bakker A, Prins J (2008). Suicidal thoughts among medical residents with burnout. Archives of Suicide Research.

[CR61] Verweij H, van Ravesteijn H, van Hooff M, Lagro-Janssen A, Speckens A (2018). Mindfulness-based stress reduction for residents: A randomized controlled trial. Journal of General Internal Medicine.

[CR62] Wallace JE, Lemaire JB, Ghali WA (2009). Physician wellness: A missing quality indicator. Lancet (london, England).

[CR63] West CP, Dyrbye LN, Erwin PJ, Shanafelt TD (2016). Interventions to prevent and reduce physician burnout: A systematic review and meta-analysis. Lancet (london, England).

[CR64] West CP, Tan AD, Habermann TM, Sloan JA, Shanafelt TD (2009). Association of resident fatigue and distress with perceived medical errors. JAMA.

[CR65] World Health Organization. (2019). Burn-out an “occupational phenomenon”: International classification of diseases. Retrieved March 14, 2022, from https://www.who.int/news/item/28-05-2019-burn-out-an-occupational-phenomenon-international-classification-of-diseases

